# Malaria infection does not affect the sensitivity of peripheral receptor neurons in *Anopheles stephensi*

**DOI:** 10.1186/1756-3305-6-134

**Published:** 2013-05-04

**Authors:** Alan J Grant, Marc A T Muskavitch, Robert J O’Connell

**Affiliations:** 1 Department of Immunology and Infectious Diseases, Harvard School of Public Health, Harvard University, 665 Huntington Avenue, Boston, Massachusetts 02115, USA; 2 Biology Department, Boston College, 140 Commonwealth Avenue, Chestnut Hill, Massachusetts, 02467, USA; 3 University of Massachusetts Medical School, Worcester, Massachusetts, 01505, USA

**Keywords:** *Anopheles stephensi*, *Plasmodium berghei*, Infection, Carbon dioxide, 1-Octen-3-ol, Electrophysiology

## Abstract

**Background:**

Mosquitoes transmit many important diseases including malaria, dengue and yellow fever. Disease transmission from one vertebrate host to another depends on repeated blood feedings by single mosquitoes. In order for the mosquito to acquire the blood that it needs to complete oogenesis, the insect must locate a suitable host. Olfactory cues (including carbon dioxide) released by the host and detected by the mosquito are the primary signals that vector insects use for host location. Previous studies have suggested that the physiological status - including bacterial, fungal, viral and *Plasmodium* infections - can modulate aspects of behavior in haematophagous insects.

**Methods:**

Standard electrophysiological techniques were used to record extracellular responses from the receptor neurons located in sensilla found on the maxillary palps of the insects. The recording microelectrode was inserted through the cuticle at the base of an individual sensillum and the extracellular electrical signals obtained from the three neurons within the sensillum were recorded. Stimulations consisted of 2 s pulses of the desired concentrations of CO_2_ or dosages of 1-octen-3-ol.

**Results:**

Accordingly, we were interested in determining whether *Plasmodium* infection affects the sensitivity of those peripheral olfactory sensors that are involved in host-seeking in mosquitoes. Our studies indicate that infection of female *Anopheles stephensi* with *Plasmodium berghei* does not alter the response characteristics of the neurons innervating the maxillary palp sensilla that respond to the attractants carbon dioxide and 1-octen-3-ol. Although the response characteristics of the peripheral sensory neurons are not affected by infection status, we found that the age of the mosquito alone does affect the threshold of sensitivity of these neurons to carbon dioxide. The proportion of older insects (21–30 d post-emergence) that responds to 150 ppm carbon dioxide is higher than the proportion that responds among younger insects (1–10 d post-emergence).

**Conclusions:**

*Anopheles stephensi* infected with *Plasmodium berghei* exhibit sensitivities to stimulation with carbon dioxide and 1-octen-3-ol similar to those of uninfected mosquitoes. However, the age of the infected or uninfected mosquito does affect the threshold of sensitivity of these neurons to carbon dioxide.

## Background

Malaria is a devastating disease caused by the protozoan *Plasmodium*, a pathogen with a life cycle that alternates between two obligate hosts: a vertebrate host (e.g., human) and an invertebrate mosquito (e.g., *Anopheles* spp.). It is during blood-feeding that an infected mosquito can potentially transmit *Plasmodium* spp. to the vertebrate host. Conversely, an uninfected mosquito can obtain *Plasmodium* spp. during feeding from an infected vertebrate host. Olfactory signals released by the vertebrate host and detected by the mosquito are integral to the host-seeking behavior of vector mosquitoes [1]. The importance of carbon dioxide (CO_2_) for mosquitoes was first reported in 1922 [2,3], and CO_2_ is generally considered to be the most important sensory cue modulating host-seeking behavior. CO_2_ is a primary by-product of cellular respiration and therefore is released by host organisms in large quantities (30,000 ppm human tidal respiratory concentration) [4]. To detect this signal, mosquitoes, as well as many other biting insects, are equipped with an array of sensors (sensilla), many of which are innervated by multiple sensory neurons. One of these neurons is highly sensitive and specifically tuned to respond to behaviourally relevant concentrations of CO_2_[5-8]. Another neuron in this sensillum responds to stimulation with low concentrations of the mosquito attractant R-(-)-1-octen-3-ol (octenol) [5,9].

Host-seeking behavior of *Plasmodium*-infected mosquitoes differs from that of uninfected mosquitoes [10,11]. Additionally, studies indicate that infection of mosquitoes with a pathogen such as a fungus, bacterium, virus or *Plasmodium* can affect various aspects of mosquito behavior. These include locomotive behavior [12,13], host-seeking behavior [14], biting and probing behavior [15,16], blood-feeding behavior [11,17-19], mating behavior [20] and fecundity [17]. Infection also increases overall mortality in mosquitoes [10,11]. Many of these behavioral changes could be influenced by olfactory inputs. Studies of *Aedes aegypti* infected with Sindbis virus suggest that infection can reduce the effectiveness of the mosquito repellent DEET [21,22]. Since it is suspected that DEET acts by modulating the octenol receptor, it is possible that the peripheral olfactory system may be involved in the behavioral changes associated with this infection. Recent work with *Ae. aegypti* also suggests that dengue infection modulates blood-feeding by acting on genes associated with chemosensory reception [23]. However, in all cases the precise mechanisms underlying these changes are not well understood. Taken together, these studies prompted us to ask whether any of the infection-induced behavioral changes might result from modulation of the sensory system.

This report asks: does infection change the sensitivity of the peripheral sensory neurons of the mosquito? To address this question, we investigated *An. stephensi* infected with *Plasmodium berghei*. We compared the CO_2_ and octenol sensitivity of uninfected and infected animals at several points during the mosquito life cycle. Infected mosquitoes were examined at 10 days post-infection and 20 days post-infection. These ages were chosen for evaluation because oocysts will be present in the midgut at 10 days post-infection and sporozoites will be present in the salivary glands at 20-days post-infection, when they are available to be transmitted to a bitten host [24].

## Methods

### Insects

*An. stephensi* were initially obtained from Dr. Maria M. Mota (Instituto de Medicina Molecular, Lisbon, Portugal). The colony was maintained in a Harvard School of Public Health insectary at 26°C, 75% RH under 12:12 L:D cycles. Infected mosquitoes and associated uninfected control mosquitoes were maintained at 20°C and 70% RH under 12:12 L:D. To obtain eggs, female mosquitoes were blood-fed using a water-jacketed membrane feeder or with a live mouse.

### Plasmodium infection

Infection was accomplished by feeding females on *P. berghei*-infected mice. *Plasmodium*-infected mosquitoes were held at 21°C and 75% RH under 12:12 L:D. Control mosquitoes (for the infection studies) were blood-fed with uninfected human blood using a membrane feeder or on an uninfected mouse and evaluated at the same ages as the *Plasmodium*-infected insects.

### Electrophysiology

Standard electrophysiological techniques were used to record extracellular responses from the receptor neurons located in the palpal sensilla found on the insects. Details of the recording methodology for mosquito sensilla have been described fully in previous reports [25-27] and are briefly summarized below. The insect was immobilized to allow unobstructed access of the recording microelectrode to the selected sensillum viewed at 750 × magnification. The recording microelectrode (electrolytically-sharpened tungsten wire) was inserted through the cuticle at the base of an individual sensillum. The indifferent electrode was placed in the eye. Microelectrodes were held in high gain, low drift micromanipulators for positioning. Electrical signals obtained from the three neurons within the sensillum were band-passed filtered (300-3 K Hz), amplified (1000 ×) and analyzed with Autospike (Syntech, Hilversum, The Netherlands). The recording set-up was housed in a Faraday cage, and the electrical circuits required for this apparatus were isolated and independently earthed through a dedicated ground circuit.

### Olfactory stimulation

Stimulation of olfactory receptor neurons was accomplished with two opposing gas streams, each directed toward the sensory structure. One of these airstreams was normally on and carried a background pure air current (550 ml/min). The second stream was normally off and carried the desired stimulus stream (440 ml/min). This was the default condition during which no recordings were obtained. Initiation of the trial and the beginning of data collection started with activation of the stimulus stream. However, since the slightly higher velocity background stream remained on for four seconds defining a prestimulus period, during which basal neural activity was recorded while the stimulus stream was effectively prevented from reaching the preparation. Following this four-second prestimulus period, the background air stream was shut off for two seconds, allowing the previously activated stimulus stream to reach the preparation. This air current stimulation protocol delivers a relatively uniform, square wave stimulus pulse to the preparation rapidly, without the delays inherent in simple sequencial valve operation. The background stream consisted of certified CO_2_-free synthetic air (AirGas, Radnor, PA), while the stimulus stream consisted of synthetic air with a calibrated concentration of CO_2_ (0, 150, 300, 600 or 1000 ppm).

Stimulus protocols consisted of 2 s pulses of CO_2_, which were presented sequentially for each of the certified concentrations, with a 14 second interval between each individual stimulation. After a rest period of five minutes, the five-concentration series was repeated. Three repetitions of this series were made for each preparation and averaged. T-tests were then performed on the grand average responses across all preparations at each dose, to determine whether there were any statistically significant differences between treatments.

### 1-octen-3-ol stimulation

We also evaluated the response to stimulation with R-(-)-1-octen-3-ol. The compound was serially diluted in light mineral oil to concentrations of 0.0001, 0.001 or 0.01 μg/μl. One μl of each dilution was applied to a filter paper strip (1.9 × 0.64 cm Whatman, #1) held in a 11.4 cm disposable glass pipette. A new paper and pipette were used for each stimulation. Stimuli were delivered by passing a 0 ppm CO_2_ stream through the pipette and against a background stream of 0 ppm CO_2_ using the same temporal sequence as was used with CO_2_ stimulation. Following each pulse of odor, the preparation was exposed briefly to room air containing a relative high concentration of carbon dioxide, to confirm the presence of the CO_2_-sensitive A neuron in the recorded preparation.

### Scanning electron microscopy

Insects were air-dried and affixed to a stub with carbon conducting paint (E.F. Fullem Co., Schenectady, NY). The stubs were then sputter-coated to a total thickness of 10 nm with gold-palladium, in three separate applications. The stubs were examined at an accelerating voltage of 5 KV with a FEI Quanta 200 FET MK II instrument at the University of Massachusetts Medical Center in Worcester, MA. Images were digitally processed and stored.

### Light microscopy

Following each recording session from infected mosquitoes, the midguts were removed, stained with mercurochrome and examined under 320 × magnification to assay for the presence of oocysts. In a few cases, older infected mosquitoes were examined by bright field microscopy, in addition, to confirm the presence of sporozoites in the salivary glands. Only data from animals microscopically confirmed to be infected with oocysts were included in the analysis.

This study was conducted using procedures for maintenance of mosquitoes and infection of mosquitoes by blood feeding on *P. berghei*-infected mice as described in protocols approved by Harvard Medical Area Standing Committee on Animals.

## Results

### Morphology and response characteristics of the maxillary palp sensilla (MPS)

**Figure 1:**
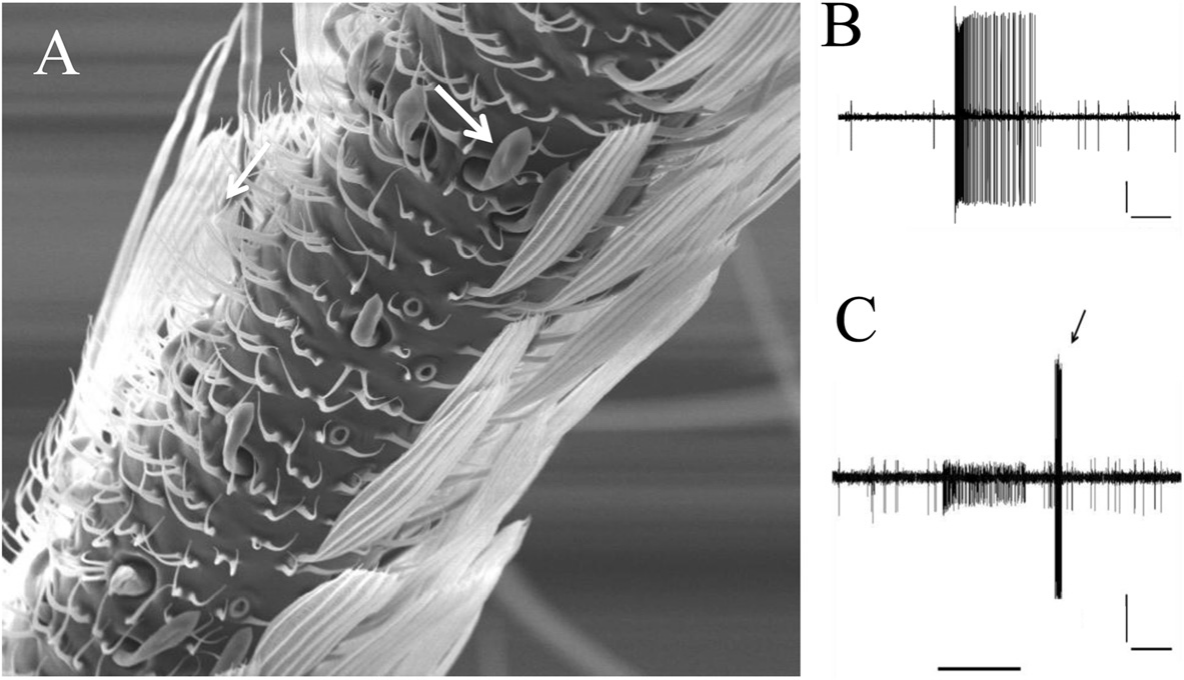
**SEM depicting several CO**_**2**_**- and octenol-sensitive maxillary palp sensilla and typical electrophysiological responses from individual sensilla to these compounds. A**. Scanning electron micrograph of the distal portion of the third subsegment of the maxillary palp from a female *An. stephensi *(6 d post-emergence) showing *s. basiconica *(arrow). **B**. Electrophysiological response from a 21 d post-emergence female *An. stephensi *to a 2 s stimulation with 300 ppm CO_2_. **C**. Electrophysiological response from a 5 d post-emergence female *An. stephensi *to a 2 s stimulation with 0.001 μg R-(-)-1-octen-3-ol. Since this recording was made in a background environment containing 0 ppm CO_2_, the **A **cell is silent. Immediately following the termination of the odor stimulus, the preparation was briefly exposed to room air, resulting in a signal burst from the A neuron (arrow). Horizontal bars under the traces in panels **B **and **C**: interval (2 s) of exposure to experimental stimulus. Verticals bars in **B **and **C** represent voltages (50 μV in **B **and 25 μV in **C**).

The bilateral maxillary palps of *An. stephensi* are approximately 1450 μm in length and contain five subsegments. The maxillary palp sensilla (Figure 1A; arrow) are distributed along the second, third and fourth of the five palpal subsegments. Each palp of *An. stephensi* contains a total of approximately 98 sensilla [28]. Each maxillary palp sensillum is innervated by three olfactory receptor neurons, each neuron producing action potentials of different amplitudes; the neuron producing the largest amplitude action potential is responsive to CO_2_ (the A neuron; Figure 1B) and the neuron producing the next largest amplitude spike (the B neuron) responds to low concentrations of the enantiomeric R form of 1-octen-3-ol (Figure 1C).

### Effects of blood feeding on sensilla sensitivity

**Figure 2:**
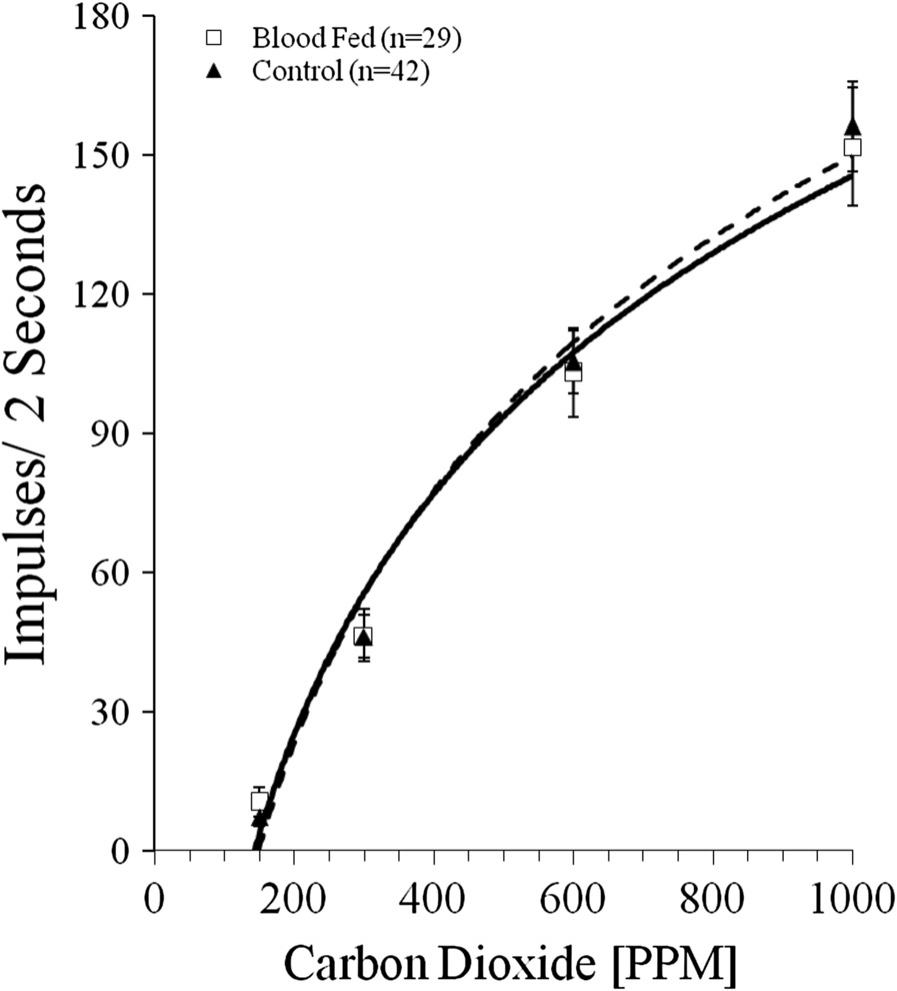
**Concentration-response relationships from uninfected blood-fed *An. stephensi *(open square, dashed line; n = 29; mean age 9.4 d) and uninfected non-blood fed control mosquitoes (solid triangles, solid line; n = 42; mean age 14.7 d). **The standard error of the mean (SEM) is indicated for each response average. Responses are not significantly different at any of the concentrations tested.

Although the primary objective of this study was to determine the potential effect of infection status on the sensitivity of the peripheral sensory system, we initially determined whether blood feeding alone can affect peripheral responses at later ages. Therefore, we compared non-infected blood-fed insects with sugar-fed insects (Figure 2). Responses to five concentrations of CO_2_ from 42 sensilla on sugar-fed insects (mean age 9.4 days) were compared to responses in 29 sensilla from blood-fed insects (mean age 14.7 days). No statistically significant differences between the two treatment groups at the ages tested were observed, throughout the range of CO_2_ concentrations assayed. We did not evaluate the effects of blood feeding on the sensory system at shorter intervals (hours or days) immediately following blood feeding.

### Effects of infection on sensillum sensitivity

#### Carbon dioxide responses

**Figure 3:**
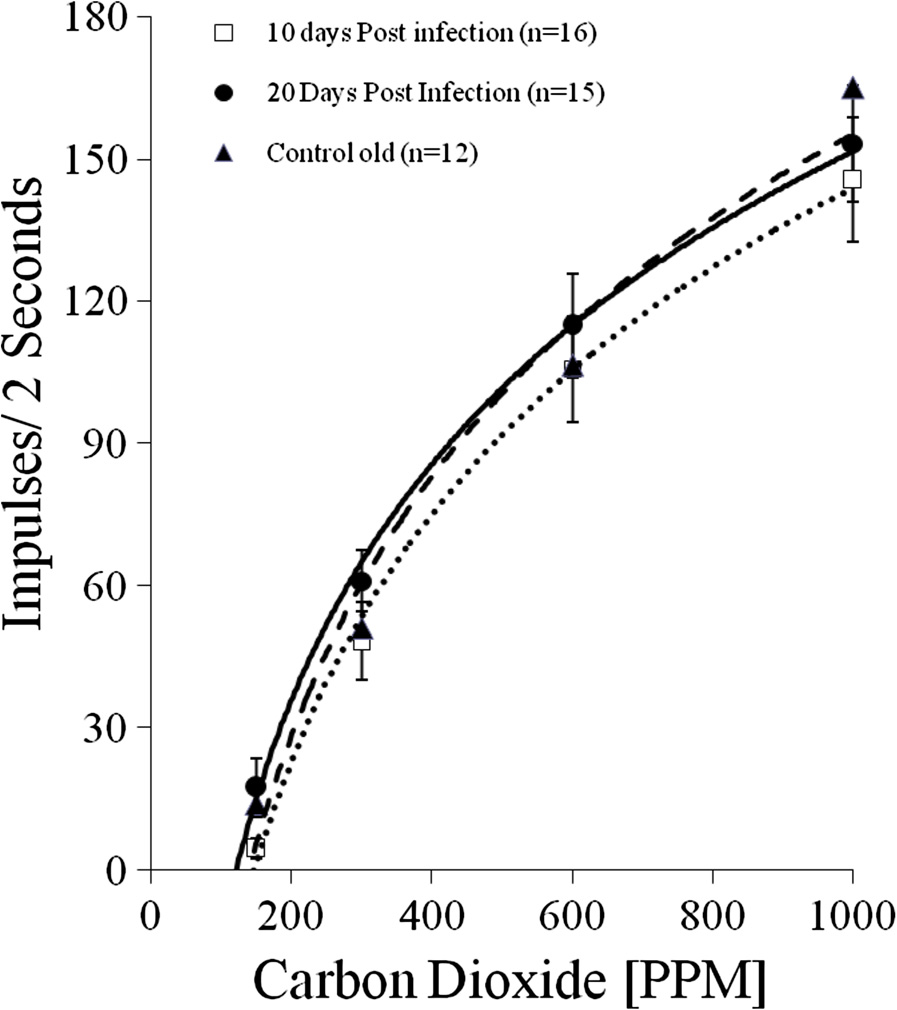
**Concentration-response relationships from 10 d post-infection female *An. stephensi *(open square, dotted line; n = 16 sensilla; mean age 18 d), 20 d post-infection female *An. stephensi *(solid circles, solid line; n = 12 sensilla; mean age 27 d) and uninfected blood-fed control female mosquitoes (solid triangle, dashed line; n = 12 sensilla; mean age 27 d). **Responses are not significantly different at any of the concentrations tested. SEM indicated for each response average.

Concentration-response relationships were established for 10 d post-infection female *An. stephensi* (n = 16 sensilla; mean age 18 d), 20 d post-infection female *An. stephensi* (n = 12 sensilla; mean age 27 d) and compared to those for uninfected blood-fed female mosquitoes (n = 12 sensilla; mean age 27 d) (Figure 3). Responses for infected and uninfected mosquitoes were not significantly different at any of the CO_2_ concentrations tested.

#### R-(-)-1-octen-3-ol responses

**Figure 4:**
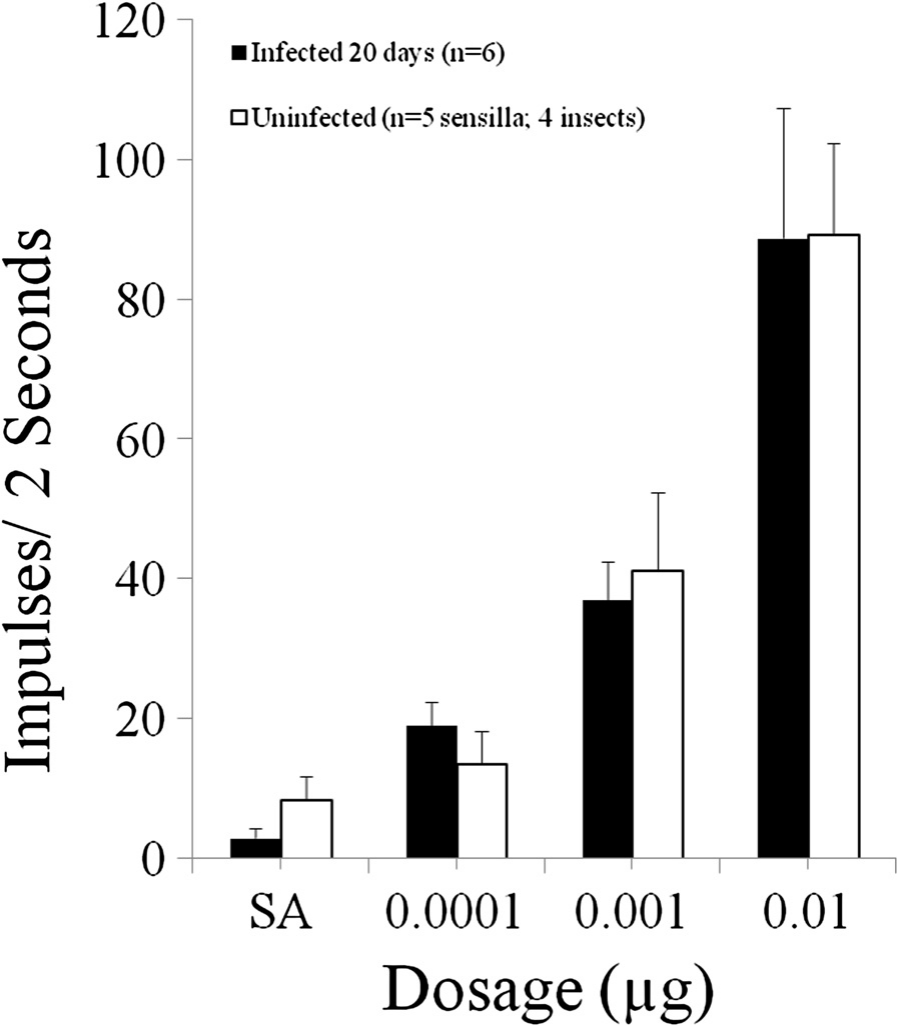
**Histogram depicting responses from infected (solid; n = 6) and uninfected (open; n = 5) maxillary palp sensilla from female *An. stephensi *that were stimulated with three concentrations of R-(-)-1-octen-3-ol.** Testing was carried out in a background environment of 0 ppm CO_2_. There was no statistical difference between the infected and uninfected insects in the mean number of spikes generated in response to stimulation at any of the three doses (0.0001 μg, 0.001 μg and 0.01 μg) of R-(-)-1octen-3-ol. SEM is indicated for each response average. “SA” is the unstimulated spontaneous activity level as recorded at the beginning of each stimulation period.

Six sensilla from five infected *An. stephensi* were simulated with R-(-)-1-octen-3-ol. The mean age of the infected insects was 27 d post-emergence, and the mosquitoes were 19.7 d post-infection at the time of testing. Five sensilla from a total of four uninfected *An. stephensi* were tested. The mean age of the control mosquitoes was 6 d post-emergence at the time of testing. Responses to stimulation with three doses of R-(-)-1-octen-3-ol (0.0001 μg, 0.001 μg and 0.01 μg) indicated no difference in responsiveness between infected or uninfected mosquitoes (Figure 4).

### Effects of age on sensilla sensitivity

**Figure 5:**
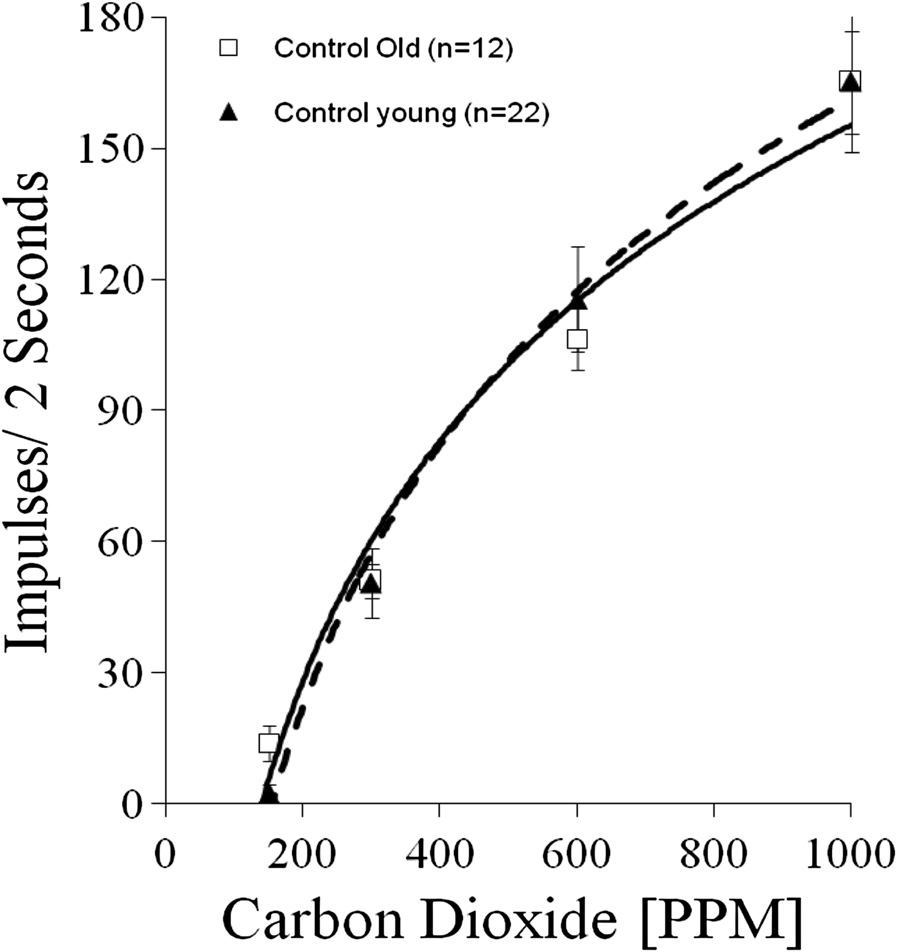
**Concentration-response relationships from young (1–10 d old) (solid triangle, dashed line; n = 22) and older (21–30 d old) (open square, solid line, n = 12) non-infected female *An. stephensi *to stimulation with CO**_**2**_**.** Responses are only significantly different at the 150 ppm concentration (p = 0.02).

Since the physiological states of the insect can change with the age of the animal, it was necessary to address the additional question: does the age of the mosquito affect the sensitivity of neurons to CO_2_? We recorded from 42 maxillary palp sensilla on 32 female *An. stephensi*. All of these mosquitoes lacked blood-feeding experience and consequently were not infected with *Plasmodium*. The 42 recordings were divided into three distinct age groups. “Young mosquitoes” were 0 to 10 d post-emergence (n = 22 sensilla from 19 insects). “Middle-aged mosquitoes” were 11 to 20 d post-emergence (n = 8 sensilla from 7 insects). “Older mosquitoes” were 21 to 30 d post-emergence (n = 12 sensilla from 6 insects). At the 150 ppm CO_2_ concentration, responses in the “young mosquitoes” were significantly different (two-tailed t-test; p = 0.02) from those of the “older mosquitoes.” Responses were not significantly different between the two age groups for any other CO_2_ concentration tested. Figure 5 presents the CO_2_ concentration-response curves for the “younger” and “older” insects. We also calculated the percentage for which there was any response to the 150 ppm stimulation in each of the three age categories for uninfected mosquitoes.

**Figure 6:**
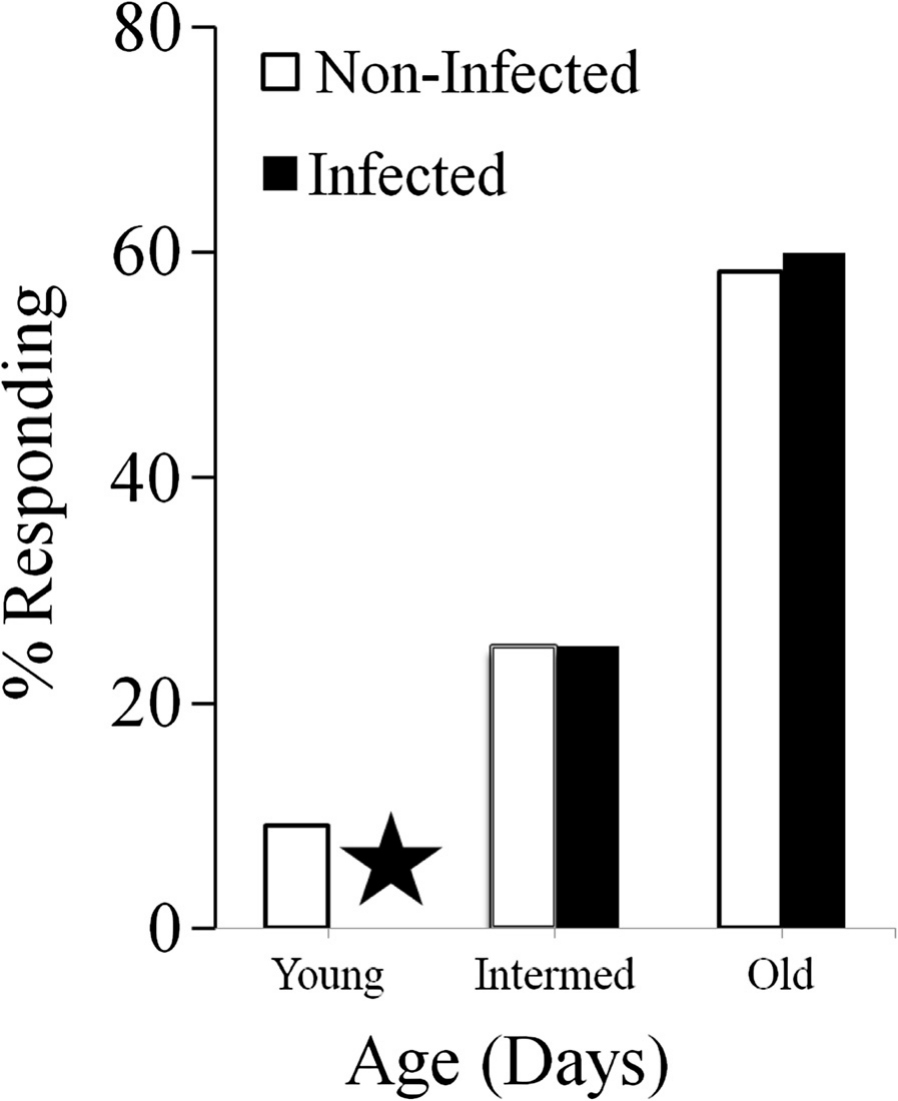
**Percentage of mosquitoes of different age cohorts that produced a response during a 2 s stimulation with 150 ppm CO**_**2**_**. **Histograms represent the percentage of those that responded at 150 ppm stimulation (solid; infected) (open; non-infected). ★ indicates that we did not determine the percentage for young infected mosquitoes showing a response since it is technically difficult to determine the infection status of these young mosquitoes, prior to the presence of midgut oocysts.

In a similar manner, we calculated the percentages responding in the “middle-aged” and “older” mosquitoes for infected mosquitoes. Data depicting the percentage of insects responding at 150 ppm for both infected and uninfected mosquitoes are presented in Figure 6. As can be seen in the histogram, responses from uninfected mosquitoes show a progressive increase in the percentage responding from “younger” to “older.” Since it is difficult to determine the infection status of young mosquitoes, we only compared “middle-age” and “older” mosquitoes for infected mosquitoes. As with uninfected mosquitoes, the “older” infected insects showed a higher percent responding than the “middle age” infected insects.

## Discussion

It is clear that mosquitos possess a sophisticated olfactory system, the outputs of which can drive orientation behavior [29]. Attraction to the volatiles emanating from a vertebrate host is a potent driver of behavior. But, obtaining blood from an active host exposes the insect to risk [30,31], so it follows that host-seeking behavior has evolved to balance the benefits and risks associated with the blood-feeding behavior. In other words, it is reasonable to expect that the sensitivity and specificity of CO_2_ receptor neurons would be optimized. The age of the mosquito and the physiological state of the insect can modulate host-seeking behavior [14,32]. If *Plasmodium*-infected mosquitoes have a more sensitive peripheral sensory apparatus, this could lead to more efficient host-location and blood-feeding. This change in sensory sensitivity could, in turn, result in a more efficient transfer of the parasite between vector and host. In most cases, we do not know the precise mechanisms that underlie these modulations. Due to the long co-evolutionary history between insect vectors and their hosts, complex co-adaptive interactions between these species may have occurred, especially with regard to semiochemicals produced by hosts and detected by insects.

Mosquitoes may be infected by a variety of pathogens, some of which can be transmitted to alternative hosts. These pathogens include viruses, bacteria, fungi and protozoans such as *Plasmodium*. Several reports indicate that various aspects of mosquito behavior can be modulated by infection status [21,22]. Relatively simple behaviors, such as overall activity level or basal metabolic rates, and more complex behaviors, such as host-seeking or blood-feeding, can be altered by mosquito infection status. Although it is clear that most behaviors are multistep processes, many of the mechanisms involved in eliciting particular vector behaviors are unknown or poorly defined. However, since much of an insect’s behavior can be mediated by olfactory signals, it seemed reasonable to postulate that one way to mediate behaviors would be to alter the peripheral sensory capabilities of the insect. Thus, behavior could be enhanced or suppressed as the sensitivity of the responsible receptor is regulated up or down.

Previous work has demonstrated that blood feeding alone can modulate host-seeking responses [33-35]. Mosquito midgut distention produced by blood-feeding leads to the release of peptides, which in turn reduces the sensitivity of lactic acid-sensitive receptor neurons on the antenna [34,36]. Although these changes would provide a reasonable sensory mechanism to account for inhibition of host seeking following blood-feeding, data presented here do not reveal a reduction in the sensitivity of CO_2_ detection by the maxillary palp sensilla after blood-feeding. The lack of effect of blood-feeding or infection on maxillary palp sensilla CO_2_ sensitivity may simply reflect a fundamental difference between the lactic acid-sensitive neurons on the antenna and the carbon dioxide sensors on the maxillary palps. Recent work with *Ae. aegypti* has indicated that specific peptides transferred from the male to the female during mating can also influence host-seeking behavior in the female [37]. We should note that the relatively long post-blood feeding intervals were chosen so that we could directly compare these data with the post-infection responses (10 and 20 days). It is possible that blood-feeding alone may indeed have effects on sensory sensitivity at intervals closer to the blood feeding event.

Our data indicate that infection of *An. stephensi* with *P. berghei* does not affect the sensitivity of maxillary palp sensilla of female *An. stephensi* with regard to the detection of carbon dioxide or octenol. This is the case for receptor neurons in mosquitoes 10 d post-infection, when oocysts are present in the midgut, and at 20 d post-infection, when sporozoites are present in the salivary glands. This lack of effect from infection on the peripheral sensory system suggests that the behavioral changes that do occur in conjunction with infection must reflect specific modifications through other sensory inputs or through central nervous system processes. It should be noted that some of the behavioral changes observed in infected mosquitoes are associated with high-intensity infections [15], which could lead to a general decline in the overall health of the insect rather than the modulation of a specific sensory mechanism by the pathogen. However, other studies suggest more specific modes of action [23].

Although the data presented here indicate that infection status does not affect the CO_2_-sensitive peripheral sensory neurons in maxillary palp sensilla, analysis of animals at different ages does suggest that the age of the mosquito can modulate peripheral sensitivity. There is a slight but significant shift in the threshold level responses to CO_2_ stimulation with increasing age. Age-related differences in peripheral sensitivity have been reported previously [9,38]. In these earlier studies, very young *Ae. aegypti* (less than 5 days old) exhibited a reduced sensitivity to CO_2_-stimulation. In this current study with *An. stephensi*, we note an age-related increase in CO_2_ sensitivity in older insects (21–30 d post-emergence). These data suggest that older insects are more sensitive to lower concentrations of CO_2_, with an increasing percentage responding to the 150 ppm CO_2_ concentration as the insect ages. The significance of this change in sensitivity is unclear; however, its implications for blood-feeding and pathogen transmission could be important if it affects activation or host orientation. Since development of infectious sporozoites requires a prolonged extrinsic incubation period in the mosquito, older mosquitoes are more infectious than younger insects. Having a more sensitive peripheral sensory receptor system might increase the mosquito’s ability to efficiently locate a host, and could consequently promote pathogen transmission.

Host-seeking and blood-feeding behaviors are absolutely crucial steps in the transmission of the malaria parasite; and as such, they represent potential control points for reducing the spread of vector-transmitted diseases. Clearly, CO_2_ and octenol are two of the important signals driving host location behavior. However, the fact that we do not observe any modification of the sensitivity of peripheral sensors to carbon dioxide or octenol as a consequence of infection does not mean that other sensory inputs are similarly unaffected. The sensitivity change associated with age [[38] and the present study] suggests that this peripheral arm of the sensory system is more dynamic than generally assumed. These findings may have implications for control methodologies in which systems utilizing the attractive nature of carbon dioxide could be tuned to attract, repel or disorient older, potentially-infected mosquitoes. Recent work by Turner *et al.*[39,40] suggests that prolonged activation of the CO_2_ sensors can lead to behavioral disorientation.

## Conclusions

The research community has thoroughly invested much time and resources into efforts aimed at vaccine and therapeutic drug development to better control malaria-associated morbidity and mortality [41,42]. Arguments can be made that due to the obligatory role of mosquitoes in the transmission of malaria, as well as other infectious diseases, investments that lead to a more thorough understanding of host-orientation behaviour and the involvement of the peripheral sensory system in such behaviour will lead to the development of additional interventions that could help control this devastating disease.

## Abbreviations

CO2: Carbon dioxide; Octenol: R-(-)-1-octen-3-ol; MPS: Maxillary palp sensilla; DEET: N,N-Diethyl-meta-toluamide.

## Competing interests

The authors declare that they have no competing interests.

## Authors’ contributions

MATM and RJO assisted in the data analysis and writing of the manuscript. AJG conceived, designed and implemented the experiments, as well as analysis of the data and writing of the manuscript. All authors read and approved the final version of the manuscript.
